# Primary gastroduodenal tuberculosis complicated with acute pancreatitis: a rare case report and literature review

**DOI:** 10.1186/s40001-020-00468-5

**Published:** 2020-12-07

**Authors:** Yuanhua Li, Suhuan Liao, Haijun Zuo, Wei Yang, Di Jiang

**Affiliations:** Department of Gastroenterology, Tungwah Hospital of Sun Yat-Sen University, Dongguan, Guangdong China

**Keywords:** Tuberculosis, Gastroduodenal tuberculosis, Endoscopy, Biopsy, Case report

## Abstract

**Background:**

Tuberculosis (TB) is a major health problem worldwide. Even in highly prevalent countries, primary gastroduodenal tuberculosis is a rare manifestation of extrapulmonary tuberculosis. In recent years, as the incidence of tuberculosis has increased year by year, the occur of gastroduodenal tuberculosis has also increased. Endoscopy is an important tool for diagnosing gastroduodenal tuberculosis. The performance of gastroduodenal tuberculosis under endoscopy is often non-specific, which may imitate other benign or malignant gastroduodenal diseases. Diagnosis of gastroduodenal tuberculosis relies on a combination of endoscopy and guided biopsy.

**Case presentation:**

Here, we report a rare and interesting case of gastroduodenal tuberculosis with acute pancreatitis. The case initially mimicked gastroduodenal ulcers in morphology and appeared in a middle-aged person with normal immunity but with prolonged fever and abdominal pain. The disease was diagnosed through endoscopy and guided biopsy, and it responded well to antituberculosis drugs.

**Conclusions:**

Clinicians must remember that even in the absence of immunodeficiency, as in this case, tuberculosis can affect any part of the gastrointestinal tract.

## Introduction

Tuberculosis remains a major public health problem, mainly affecting low- and middle-income countries, causing more than 1.5 million deaths each year [1]. Although the most common manifestation of tuberculosis is the lung, cases of extrapulmonary tuberculosis are not uncommon, but the stomach and duodenum are rare parts of tuberculosis [2, 3]. The unique characteristics of the stomach and duodenum such as the sterilizing properties of stomach acid, lack of lymphatic tissue, rapid emptying and intact mucosa, can protect organs from tuberculosis [4]. Gastroduodenal tuberculosis is usually the result of secondary spread of primary lung disease. According to reports, the incidence of gastroduodenal tuberculosis is only 0.5%, of which primary gastroduodenal tuberculosis is even rarer [2, 3]. Due to the rarity of the disease, the clinical manifestations have no obvious specificity, and the auxiliary examination, especially under the gastroscopy, is not typical, so most of them are misdiagnosed, such as gastric ulcers, gastric cancer, and gastric submucosal tumors [5–7]. We introduce a case of primary gastroduodenal tuberculosis, and summarize the characteristics of gastroduodenal ulcer.

## Case presentation

**Fig. 1:**
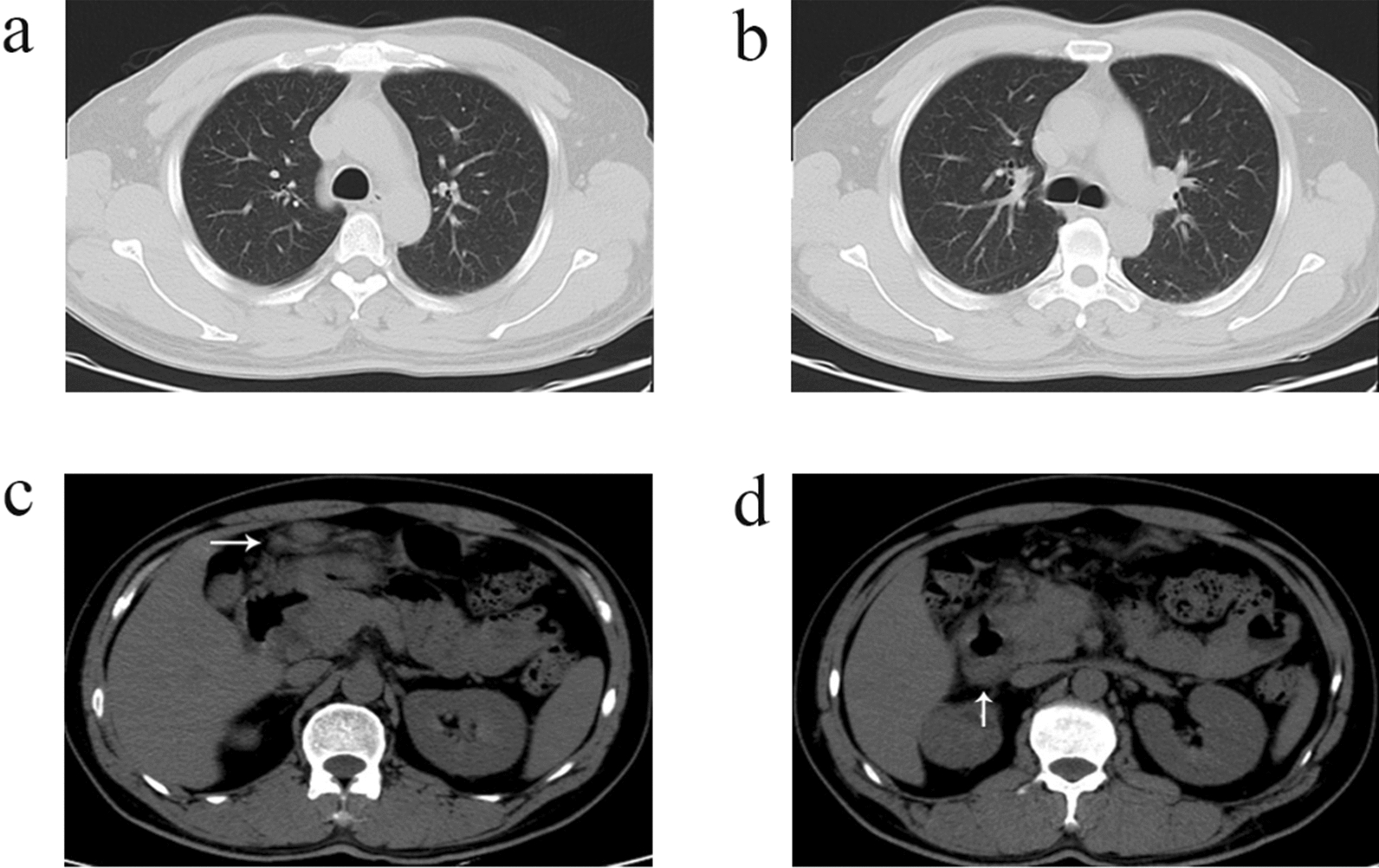
Images of chest CT and abdominal CT scan. Images **a**, **b** of chest CT showing no obvious primary tuberculosis foci in the lungs. The CT image **c** of the abdomen showing exudation and multiple enlarged lymph nodes in the antrum and duodenum. Image **d** of the abdomen CT showing uneven thickening of gastric antrum and duodenal mucosa

A 41-year-old male patient was admitted to Tungwah Hospital of Sun Yat-Sen University on April 5, 2019 due to prolonged fever and upper abdominal pain. Five days before admission, he had prolonged fever for no apparent reason. The maximum body temperature was 40 °C, accompanied by chills. The body temperature reduced to normal after taking antipyretics. He began to experience upper abdominal pain 10 h before admission. Throughout the disease, he had no cough, sputum, hemoptysis, nausea, vomiting, abdominal distension, diarrhea, chest tightness, and shortness of breath. He claimed that he had not been exposed to tuberculosis before. Outpatient chest computerized tomography (CT) showed no obvious infection lesions in the lungs (Fig. 1a,b), while chemical tests showed that amylase was 448 U/L and lipase was 1225 U/L. The patient was initially suspected of having acute pancreatitis. After admission to the hospital, CT examination of the abdomen showed that the gastric antrum-duodenum was unevenly thickened with the surrounding oozing and multiple enlarged lymph nodes (Fig. 1c,d). CT enhanced examination and electronic gastroscopy are recommended for further examination. The patient was treated for acute pancreatitis at the beginning of admission. After 6 days of treatment, his upper abdominal pain relieved, but he still had repeated fever.

**Fig. 2:**
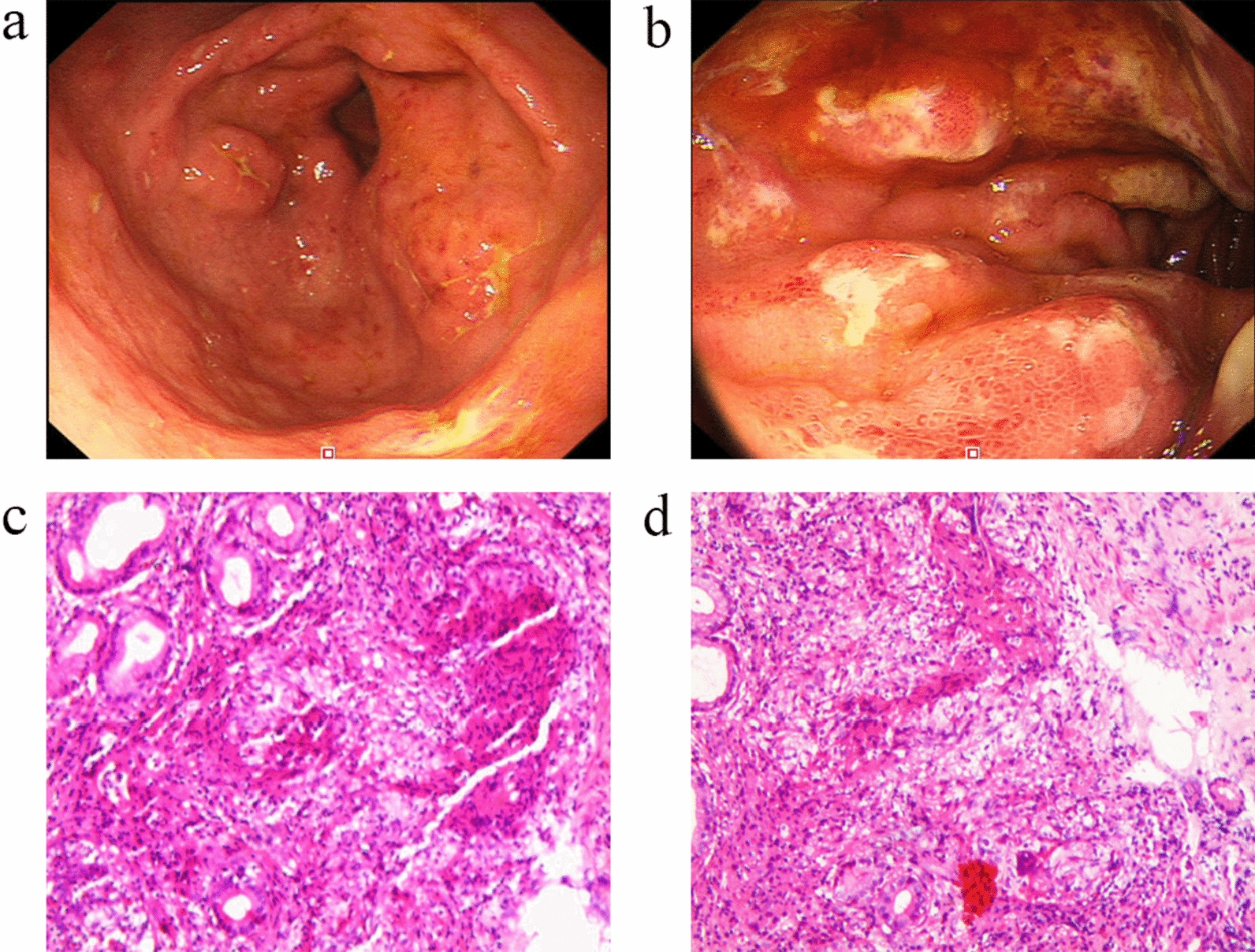
Images of gastroscopy and histological examination. Images of gastroscopy showing multiple ulcers in the gastric antrum (**a**) and mucosal edema and multiple ulcers in the duodenal bulb (**b**). Hematoxylin and eosin staining showed chronic active mucosal inflammation, granulomatous inflammation, and epithelioid granulomas in the local lamina propria (**c**, **d**) (**c** ×200, **d** ×100)

After abdominal CT results came out, we performed a gastroscopy for the patient. Gastroscopy revealed that the patient had multiple gastric and duodenal ulcers (Fig. 2a,b). After biopsy, histopathology showed that the gastric antrum mucosa was chronically active inflammation and infected helicobacter pylori, and epithelioid granulomas were seen in the lamina propria, which was in line with the ulcer marginal tissue (Fig. 2c,d). Histopathology suggested tuberculosis should be considered. To find more evidence to support the diagnosis of tuberculosis, we arranged the tuberculosis antibody test, interferon-gamma release assay (T-SPOT.TB) and purified protein derivative test (PPD skin test) for the patient. The anti TB antibody test was positive, the T-SPOT.TB result was uncertain, and the PPD skin test was negative. After excluding other pathogen infections and autoimmune diseases, we arranged an empirical anti-tuberculosis program for the patient. The patient received systematic IREZ anti-tuberculosis treatment (ATT) for 1 week (isoniazid 0.3 g, orally once a day; rifapentine 0.45 g, orally twice a week; ethambutol 0.75 g, orally once a day; pyrazinamide, orally 3 times a day), while taking glutathione tablets to protect liver. After the third day of the anti-tuberculosis treatment process, the body temperature reduced to normal without recurring fever. One week later, the patient did not develop fever or abdominal pain again and was discharged from the hospital. After the patient was discharged from the hospital, treatment continued in the outpatient clinic. During the 12-month follow-up, the patient claimed that he did not have abdominal pain, fever, or night sweats again.

## Conclusions and discussion 

Gastroduodenal tuberculosis, like tuberculosis in other parts, mostly occurs in areas with poor living conditions and underdeveloped economies [1]. Tuberculosis can occur in various parts of the gastrointestinal tract, among which the ileocecal junction is the most common, and the stomach is the rarest [4]. The case we reported is a rare multiple gastroduodenal tuberculosis. *Mycobacterium tuberculosis* invaded the stomach and duodenum, causing damage to the gastrointestinal mucosa [1]. The age of onset is mostly 20–40 years. Most gastric tuberculosis occurs in the antrum and pylorus, which are also a high incidence of peptic ulcers [8]. *Mycobacterium tuberculosis* can invade the stomach through the following ways: (1) *Mycobacterium tuberculosis* swallowed directly invades the gastric mucosa; (2) *Mycobacterium tuberculosis* in other parts invades the stomach wall through the blood system or lymphatic system; (3) *Mycobacterium tuberculosis* from nearby organs spreads to stomach. In the case we reported, the patient did not have tuberculosis in his lungs and probably swallowed *Mycobacterium tuberculosis* which caused gastroduodenal tuberculosis.

Gastroduodenal tuberculosis has no unique clinical symptoms and its clinical manifestations are similar to most gastrointestinal diseases. Sometimes gastroduodenal tuberculosis is also associated with ulcers, gastric cancer and other diseases, making the diagnosis more complicated and is easy to be misdiagnosed and missed [9]. In the case we reported, after treatment for acute pancreatitis, abdominal pain of the patient relieved, but he still had prolonged fever. The tuberculosis symptoms are easily masked by acute pancreatitis. The diagnosis of gastroduodenal tuberculosis is a challenge, because it has no specific symptoms, no specific radiological and endoscopic features, and mainly relies on gastroscopic biopsy or postoperative pathological examination. Pathological results suggesting that lesions have caseous granuloma or positive staining of acid-fast bacilli are considered to be effective clinical evidence [10]. However, even if a pathological biopsy is performed under a gastroscope, the pathological result of lesions may be non-specific inflammation [11]. Ishii et al. [12] once reported that patients with primary gastric tuberculosis showed negative acid-fast bacilli staining but were effective for empirical ATT. Tuberculous granuloma is more likely to produce negative results, which may be related to the deeper location of tuberculous granuloma [13]. In recent years, various molecular and immunological techniques and other biological techniques have also been increasingly used for rapid diagnosis of gastrointestinal tuberculosis [4]. The study by Baylan et al. [14] showed that acid-fast bacilli staining and biopsy specimens were negative for bacterial culture, while *Mycobacterium tuberculosis* was positive for polymerase chain reaction (PCR). Therefore, endoscopists must perform multiple deep resection biopsies, repeated biopsies, and other methods that are most likely to increase the detection rate of tuberculosis according to the characteristics of lesions. Anti-tuberculosis treatment is still the main measure and all cases should receive treatment for at least 6 months, and can be extended to 9–12 months if necessary. For poorly treated patients, if there are pyloric obstruction, bleeding, huge ulcers, gastric cancer, etc., surgery can be used to completely remove the lesions.

This disease is clinically rare, and lack of understanding is the main reason for misdiagnosis and missed diagnosis. Patients with multiple ulcers, pyloric obstruction, upper abdominal masses, gastrointestinal bleeding and other symptoms, especially patients with primary tuberculosis and bone tuberculosis, should undergo detailed and systematic examination. Clinicians should improve their understanding of various manifestations of gastric tuberculosis and become familiar with its diagnosis. When gastric tuberculosis is suspected, biopsy should be performed in time and multiple times, and diagnostic tuberculosis treatment is feasible if necessary.

## Data Availability

Not applicable.

## Abbreviations

TB: Tuberculosis
CT: Computerized tomography
T-SPOT.TB: T cell spot test for tuberculosis infection
PPD: Purified protein derivative
ATT: Anti-tuberculosis treatment
IREZ: Isoniazid, rifapentine, ethambutol, pyrazinamide
PCR: Polymerase chain reaction
